# End-member modeling of the grain-size record of Sikouzi fine sediments in Ningxia (China) and implications for temperature control of Neogene evolution of East Asian winter monsoon

**DOI:** 10.1371/journal.pone.0186153

**Published:** 2017-10-12

**Authors:** Hanchao Jiang, Shiming Wan, Xiaolin Ma, Ning Zhong, Debo Zhao

**Affiliations:** 1 State Key Laboratory of Earthquake Dynamics, Institute of Geology, China Earthquake Administration, Beijing, China; 2 Key Laboratory of Marine Geology and Environment, Institute of Oceanology, Chinese Academy of Sciences, Qingdao, China; 3 State Key Laboratory of Loess and Quaternary Geology, Institute of Earth Environment, Chinese Academy of Sciences, Xi’an, China; Chinese Academy of Sciences, CHINA

## Abstract

The Late Cenozoic East Asian winter monsoon (EAWM) enhancement has been attributed to several factors, such as uplift of the Tibetan Plateau, retreat of the Paratethys Sea, and global cooling related to polar ice volume increment. However, the fundamental forcing factors remain enigmatic due to the absence of long and continuous climate records and sensitive indicators. Here we reanalyzed the published grain-size record of Sikouzi fine sediments in the western Chinese Loess Plateau through end-member (EM) modeling. The results indicate that EM 2 with grain-size peaks between 10–100 μm decreased in content from 20.1 to 17 Ma and stepwise increased from 17 to 0.07 Ma during the following six stages (17–15 Ma, 15–12 Ma, 12–8 Ma, 8–6 Ma, 6–4 Ma and 4–0 Ma). Such varying trends can be successively correlated in seven stages with the integrated benthic δ^18^O record, implying that global warming weakened the EAWM from 20.1 to 17 Ma and global cooling has stepwise strengthened the EAWM since 17 Ma. Therefore, we conclude that global temperature change played a major role on the evolution of EAWM during the Neogene period. By contrast, Late Cenozoic palaeogeographic reorganization caused by uplift of the Tibetan Plateau and retreat of the Paratethys Sea contributed less to the evolutionary evolution of EAWM. Spectral analysis of the EM 2 data first provided direct evidence of orbitally influenced deposition in the study area and thus the EAWM variations during the Neogene period. The 100-kyr period became weak since ~10 Ma, possibly due to the decrease in sensitivity of a more stable, continental-scale ice sheet in Antarctica to local insolation forcing, deserving further investigation.

## 1. Introduction

Dust has extensively deposited in the Chinese Loess Plateau (CLP) at least since the Late Oligocene [1–4]. Most loess deposits are generally underlain by Miocene to Pliocene red clay [4–10]. Both loess and red clay are dominated by silt particles. It is a common sense that the Quaternary loess was generally transported by the East Asian winter monsoon (EAWM) though dry riverbeds have recently been recognized as important dust sources for the CLP and, by inference, for the downwind North Pacific Ocean [11–13]. But the pre-Quaternary dust in the CLP has different interpretations. For example, grain-size records of bulk samples from four sections (Lingtai, Xifeng, Zhaojiachuan and Luochuan) in the central CLP are used to reveal variations in the EAWM and westerly circulation [9] while quartz grain size is selected as a winter monsoon index [10]. Accordingly, a new continuous sensitive record is needed to explore long-term evolution of the EAWM so that its controlling factors can be addressed.

**Fig 1:**
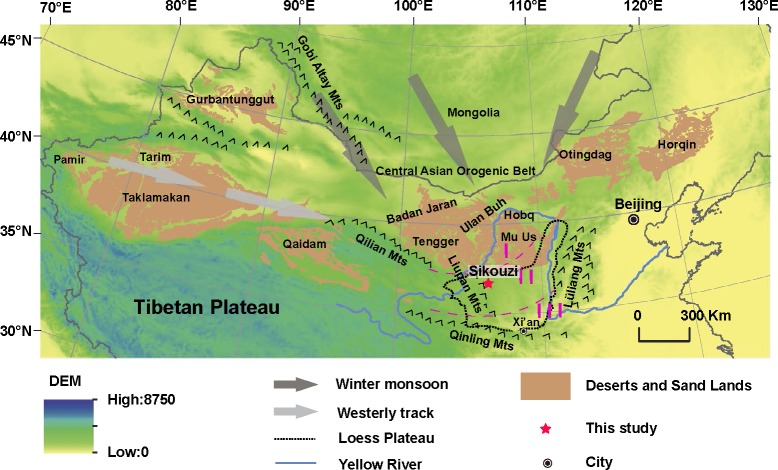
Digital elevation model (DEM) map of northern China. Zone I-sandy loess; zone II-loess; zone III-clayey loess. The decrease in loess grains from northwest to southeast is consistent with the northwesterly winter monsoon winds over East Asia. The desert and mountains are indicated (adapted from [55]).

In semi-arid to arid regions, dust particles can be easily trapped by moist surfaces including water bodies and vegetated surfaces like in a basin. A well-exposed, 2880-m-thick fluviolacustrine sequence at Sikouzi, Guyuan, Ningxia, China (Fig 1), suggested that a grand basin long-term developed in the eastern Liupan Mountains in the western CLP during the Neogene period, which was demonstrated generally continuous by magnetostratigraphic investigation, spanning from 20.1 to 0.07 Ma [14]. Rare earth element patterns and sedimentary features of representative samples from the Sikouzi sequence pointed to the windblown origin of Sikouzi fine sediments [15]. This is well consistent with recent provenance recognition of Late Cenozoic lacustrine sediments in North China [16–17]. Although our previous study presented a rough three-stage evolution of the Sikouzi grain-size record [15], detailed numerical analysis was not conducted and more information on climate change remains to be detected.

## 2. End-member modeling of the grain-size record

**Fig 2:**
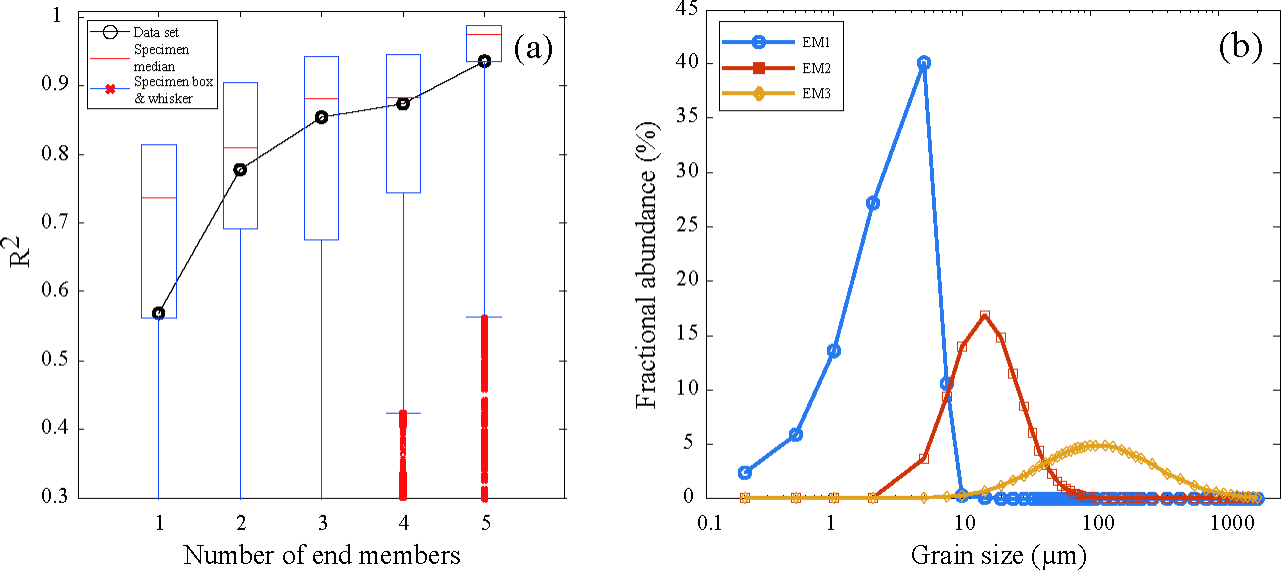
Correlation map between multiple correlation coefficient (R^2^) and number of end-member (a), and three selected end members (b).

**Fig 3:**
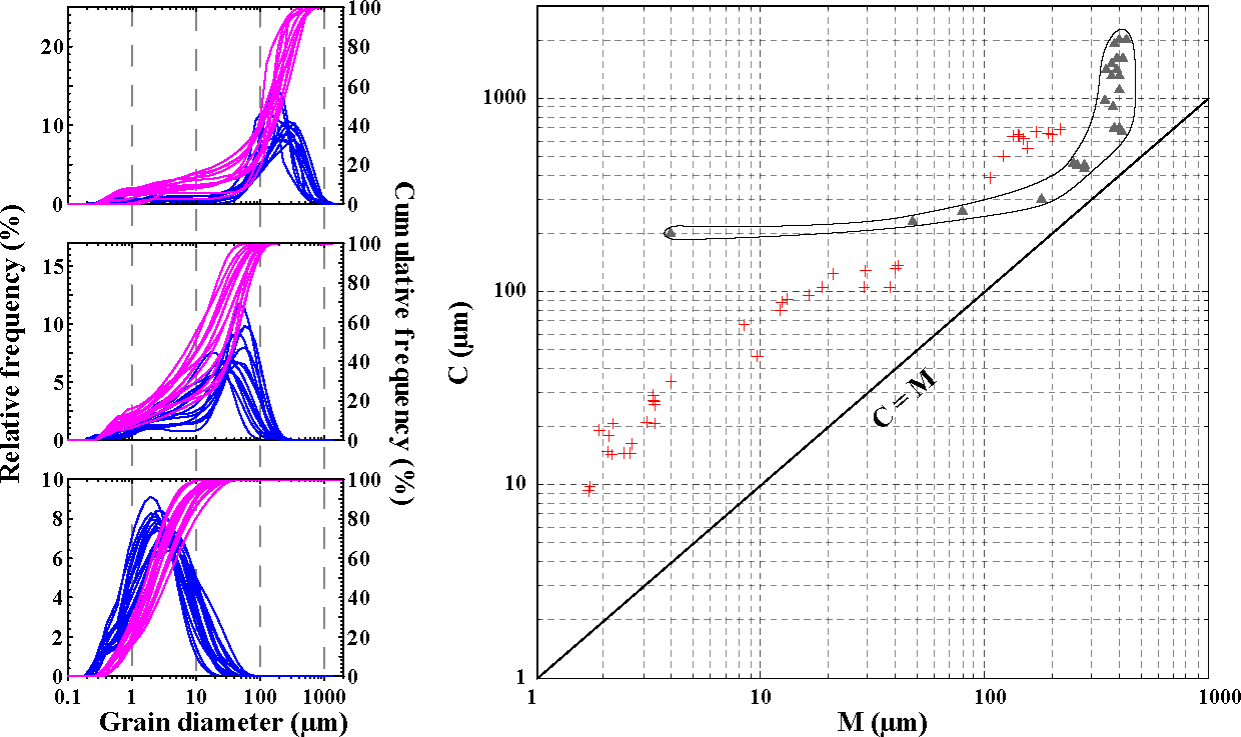
Relative and accumulative frequency of 44 representative samples selected from the Sikouzi grain-size sequence are correlative to 3 end members in Fig 2, and their distribution in a C-M plot (red cross) is in contrast with that of the Mississippi river samples (gray triangles, [31]). The former is parallel to line C = M while the latter shows an L shape.

Numerical unmixing of grain size distribution data into constituent components, known as end-member analysis (EMA), can yield valuable information on geological processes and palaeo-environmental changes [18–20]. In this study, we reanalyzed the Sikouzi grain-size data composed of 3398 samples [15] using a new developed GUI software of AnalySize for processing and unmixing grain size data [19]. In the correlation map between multiple correlation coefficient (R^2^) and end-member number (Fig 2A), end-member modeling improved greatly from 2 to 3 end members, but improved fairly less from 3 to 4 end members. Given that explaining the observed compositional variation requires a minimum number of end members in EMA [18], three end members were modeled in this study and their peak values concentrated at 1–10 μm (EM 1), 10–100 μm (EM 2), and more than 100 μm (EM 3), respectively (Fig 2B). Noticeably, the study area lies in the western CLP and remained arid to semi-arid during the Neogene period [21–22], which is supported by the spatial and temporal variations in *Fupingopollenites* percentages across Inner and East Asia [23]. Previous studies suggest that the clay mineral composition in loess and soil was of clastic origin [24] and that some clay-size material is formed in low energy aeolian environments [25] or mountain processes such as glacial grinding, frost weathering, salt weathering and even earthquakes [26–30], and thus variation in EM 1 reflected a background deposition of dust. By contrast, abundance fluctuation in EM 2 probably indicated variations in the East Asian winter monsoon (EAWM) and the EM 3 fraction probably came from nearby the study area [15]. Correspondingly, 44 representative samples of Sikouzi fine sediments were selected and divided into three groups, and their relative and accumulative frequency curves were presented in Fig 3. They seemingly reflect different dynamics of transportation.

## 3. Provenance analysis of C-M pattern

**Fig 4:**
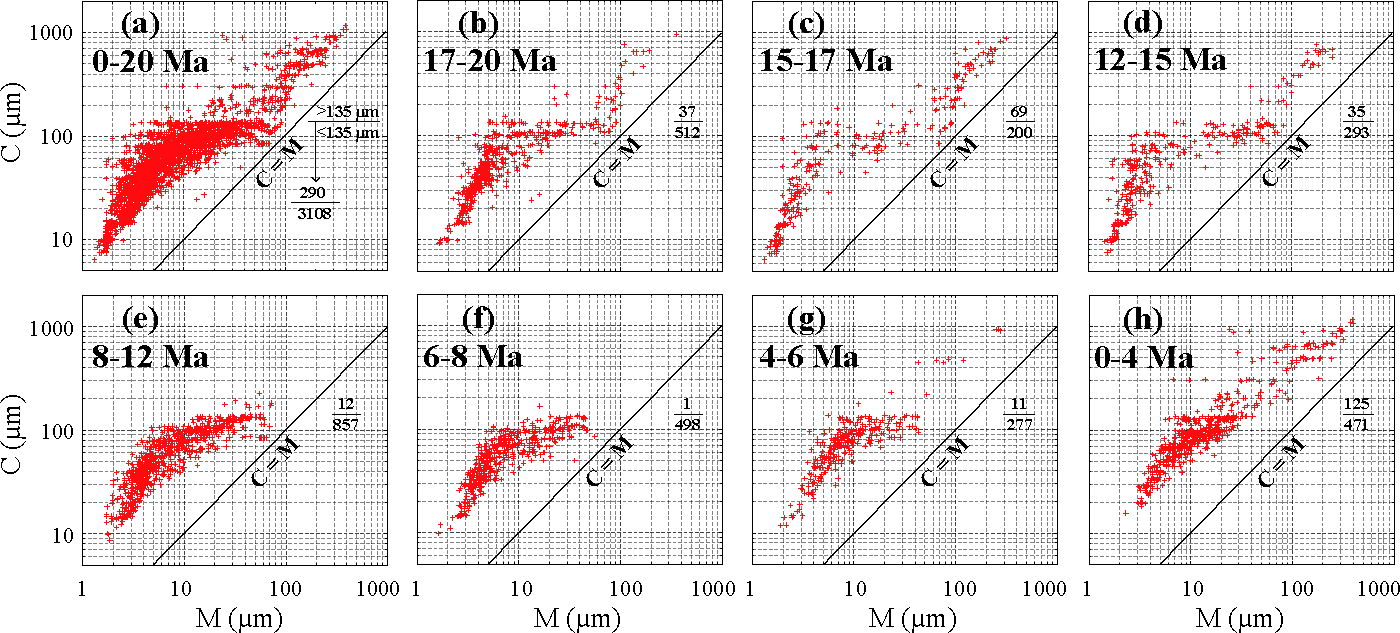
Grain size distribution of the Sikouzi fine samples in a C-M plot during past 20 Ma and during different time intervals.

C-M patterns comprised by the one percentile (C) and the median diameter (M) are characteristic of the depositional agent [31], and different parts of a C-M pattern reflect different processes of transportation and deposition [32]. We compared our 44 representative samples with the Mississippi river ones [31] in Fig 3. Distribution of the Mississippi river samples showed an L shape with knee point closest to the C = M line, indicating that only a few of river samples had a relatively good sorting. In contrast, 44 Sikouzi fine samples (Fig 3), and even almost all of the Sikouzi samples, whether the fine (C < 135 μm) or the relatively coarse (C > 135 μm) ones (Fig 4), are parallel to sub-parallel with the C = M line, implying that they had much better sorting than the Mississippi river samples [31–32]. This further supports the windblown origin of Sikouzi fine sediments [15], and is also consistent with our recent major and minor element analysis (under submission). Furthermore, C values are usually more than 200 μm for river samples and M values are often less than 10 μm for deep sea or deep lake samples [32]. These samples distribute in different areas from our samples. Thus these distribution features can readily differ the Sikouzi windblown sediments from river or deep lake ones.

Therefore, the relatively coarse ones with C values > 135 μm (only 290 in all, ~8.5% of the total 3398 samples), like the relatively fine ones with C values < 135 μm, are possibly windblown in origin as well, because they are concentrated in the CM plot and are parallel to the C = M line (Fig 4), showing a feature of good sorting for aeolian deposit. These relatively coarse particles were probably transported by ambient wind [33–35] or gust [36] from nearby sources.

## 4. Discussion

**Fig 5:**
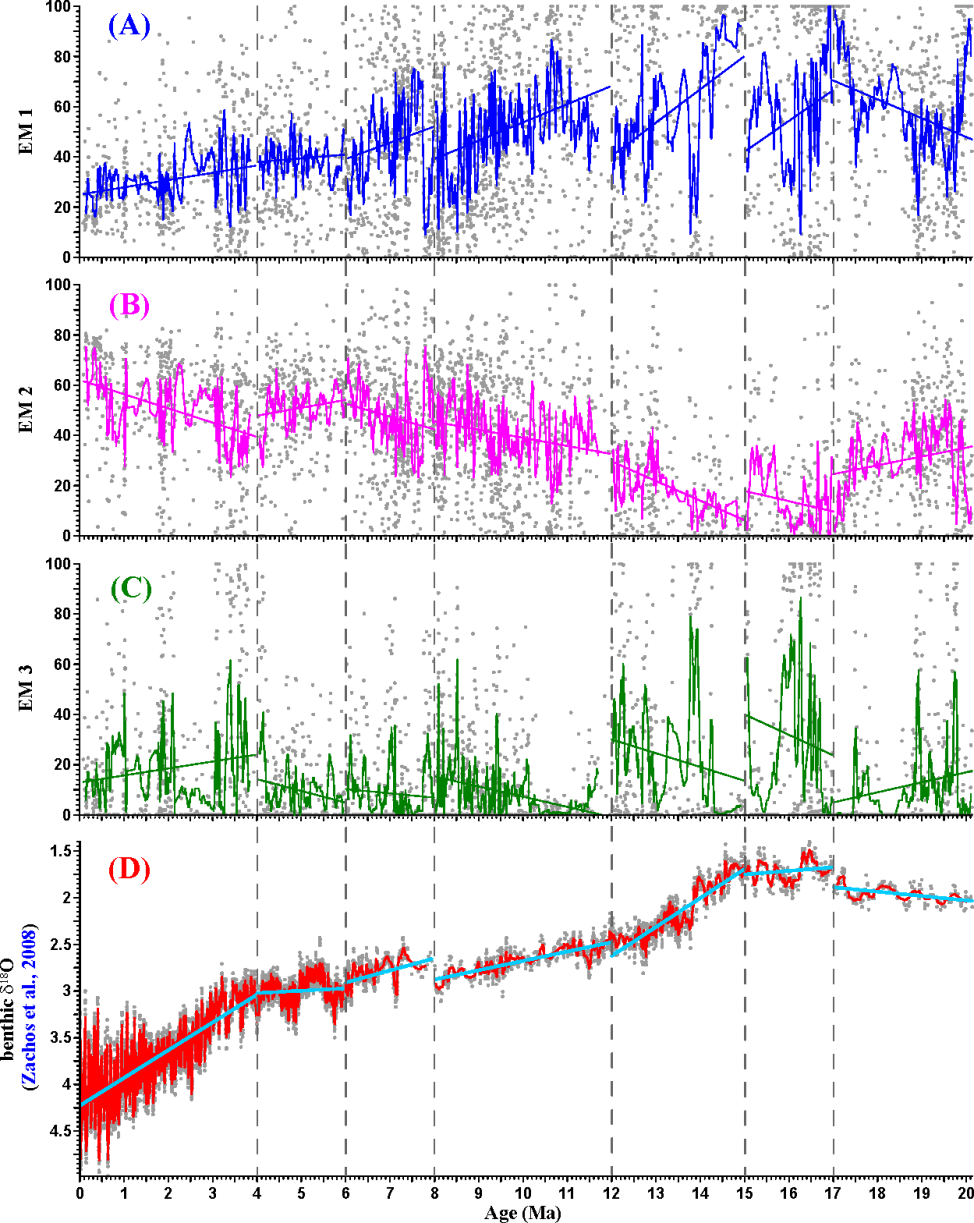
Variations of three end members of the Sikouzi grain-size sequence spanning the past 20 Ma plotted against paleomagnetic ages and its correlation with the integrated δ^18^O curve [37]. For each time interval, the solid line is linear fitting and the solid curve is averaging with a window width of 11 data points.

In this study, each of three end-members varied from zero to 100% but had different averages (Fig 5). EM 1 had a mean value of 47.4% while EM 2 had an average of 38.4%. By contrast, EM 3 had a low mean value of 14.2%. In order to present a clear varying trend for each end-member, we run a linear fitting and averaging with a window width of 11 data points for each time interval. EM 1 generally increased in abundance from 20.1 to 17 Ma and stepwise decreased from 17 to 0.07 Ma (Fig 5A). On the contrary, percentage of EM 2 decreased from 20.1 to 17 Ma and stepwise increased from 17 to 0.07 Ma (Fig 5B). Intriguingly, EM 3 also showed a similar varying trend to EM 2 except the last time interval since 4 Ma (Fig 5C).

Such clear varying trends for three end-members of the Sikouzi grain-size record in Ningxia can be well correlated with the benthic foraminiferal composite δ^18^O record [37] (Fig 5D). Given that shifts in δ^18^O are believed to reflect changes in global ice volume and thus variations in global temperature [37–39], we believe that shifts in δ^18^O were tightly associated with changes in global temperature during the late Cenozoic period. From 20.1 to 17 Ma, both EM 2 and the δ^18^O curve showed a decreasing trend, implying that global warming during this period weakened the EAWM. From 17 to 0.07 Ma, the δ^18^O curve, EM 2, and EM 3 showed a stepwise increasing trend (Fig 5), implying that global cooling stepwise strengthened the EAWM since 17 Ma. The middle-late Miocene transition and the significant development of East Antarctic Ice Sheet probably not only strengthened the meridional temperature gradients and global aridity in the middle-high latitudes, but also intensified the oceanic and atmospheric circulation and the major falling of global sea level [21–22]. Several positive feedback mechanisms possibly modulated and magnified the mid-Miocene global cooling, including vegetation changes, greenhouse gas (atmospheric CO_2_ and water vapor) fluctuations as Jiang et al. [39] proposed.

Noticeably, from 6 to 4 Ma, whether the δ^18^O curve, EM 2 or EM 1, showed a slow variation or maintained relatively stable, probably because the climate in Asia corresponded to global warming during the Early Pliocene [40–41]. Supporting this viewpoint, sea surface temperature had almost no cooling from 6 to 4 Ma in the northwestern Pacific [42].

From 4 to 0.07 Ma, both the δ^18^O curve and EM 2 showed a rapid increase to the highest values while EM 1 declined to the lowest for the whole sequence (Fig 5), indicating prominent increase in polar ice volume was responsible for significant strengthening of the EAWM over the past 4 Ma [15, 37].

**Fig 6:**
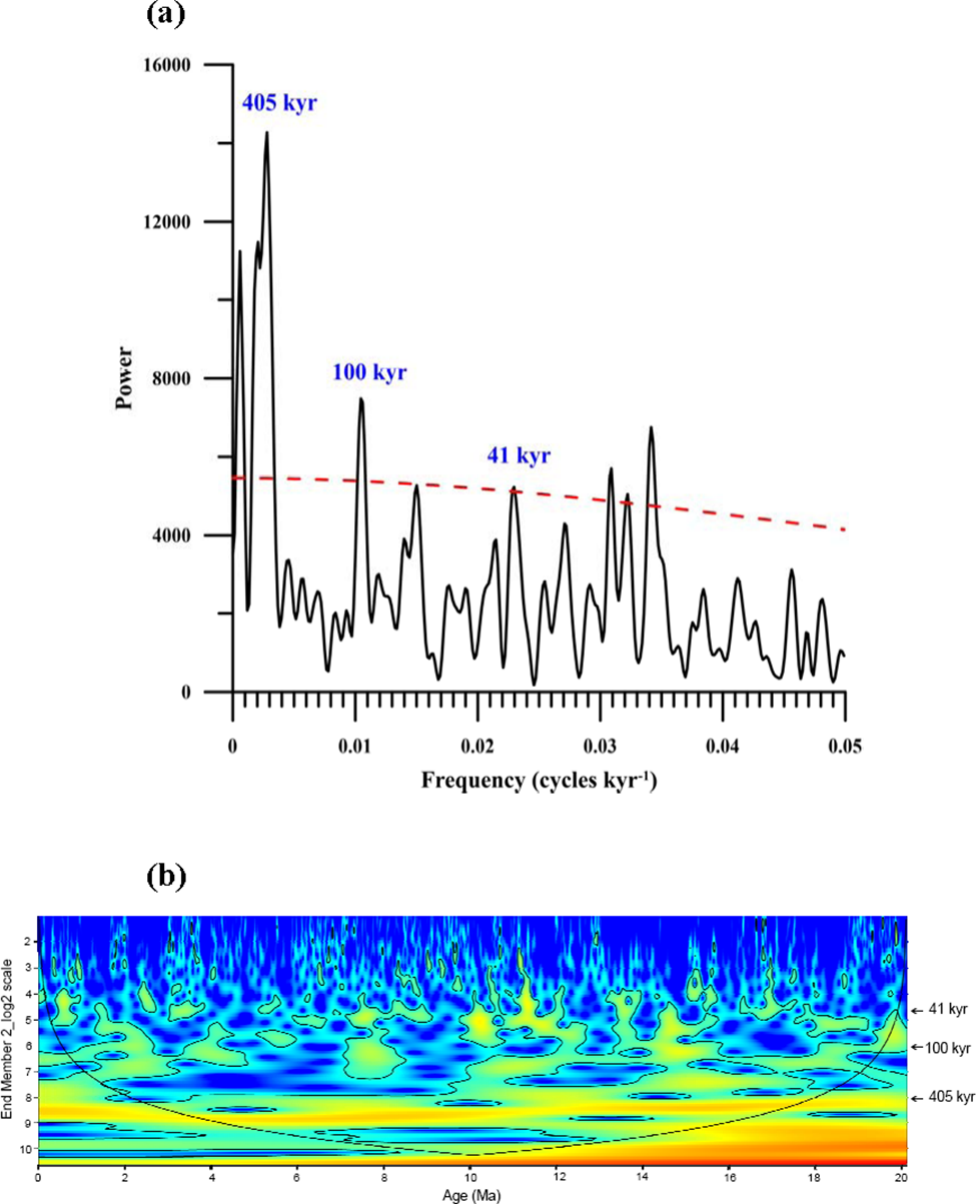
Power (a) and evolutive (b) spectrums over the past 20 Ma on the detrended EM 2 data of Sikouzi grain-size record. Note that the 405-kyr period kept strong over the past 20 Ma and the 100-kyr period weakened since ~10 Ma.

Under the age control of biostratigraphy and magnetostratigraphy [14], the EM 2 data of the Sikouzi grain-size record were detrended with a first difference filter to remove low-frequency variance. We used the REDFIT38 program [43] to analyze the EM 2 data deducted by LOESS (locally weighted scatterplot smoothing). Spectral analysis shows a clear forcing at eccentricity (405 kyr and 100 kyr) and obliquity (41 kyr) (Fig 6A), for the first time providing direct evidence of orbitally influenced fluctuating cycles of dust deposition in the study area and thus the EAWM variations during the Neogene period. Furthermore, we used the wtc-r16 Matlab package to conduct continuous wavelet analysis [44] on the detrended EM 2 data. The results show that the 405-kyr period was generally strong over the past 20 Ma and got obviously stronger during the Middle Miocene Climate Optimum (MMCO). Interestingly, during the same period, both the benthic and planktonic δ^13^C records at Site U1337 in the east equatorial Pacific reveal marked 405-kyr carbon isotope cycles [45] as in ocean carbon reservoir [46], probably indicating that the long eccentricity (405-kyr) paced carbon inputs from terrestrial weathering to ocean [45] and possibly drove the East Asian summer monsoon [47]. The 100-kyr period became weak after ~10 Ma (Fig 6B), possibly due to the decrease in sensitivity of a more stable, continental-scale ice sheet in Antarctica to local insolation forcing [48]. Noticeably, the 100-kyr period became strong at ~8.5–7 Ma, which is well correlated with the analyzed results of Late Miocene lacustrine record from the eastern Qaidam Basin in Northwest China [49], possibly due to Southern Hemisphere insolation-driven Antarctic ice sheet forcing or ephemeral variations of the Northern Hemispherie ice sheets before 7 Ma [50–51]. This inference is well consistent with the enhanced amplitude variation of the 100-kyr period during the Mi events [52], implying that the 100-kyr cycle was strengthened at times of glacial maxima as they were during the Late Pleistocene. Since ~5 Ma, the 100-kyr period showed a higher variability than before, which is probably associated with the development and fluctuation of bipolar ice volume [47, 53–54]. These deserve further investigation in the future.

## 5. Conclusion

EMA of the grain-size record of Sikouzi lacustrine sediments in Ningxia indicates that the varying trend of three end members can be successively correlated in seven stages with the integrated benthic δ^18^O record, implying that global warming weakened the EAWM from 20.1 to 17 Ma and global cooling stepwise strengthened the EAWM since 17 Ma. Hence, we conclude that global temperature related to polar ice volume played a major role on the long-term evolution of EAWM during the Neogene period. By comparison, Late Cenozoic palaeogeographic reorganization caused by uplift of the Tibetan Plateau and retreat of the Paratethys Sea contributed less to the long-term evolution of EAWM. Spectral analysis of the EM 2 data first provided direct evidence of orbitally influenced deposition of dust particles in the study area and thus the EAWM variations during the Neogene period. The 100-kyr period weakened since ~10 Ma, possibly due to the decrease in sensitivity of a more stable, continental-scale ice sheet in Antarctica to local insolation forcing, deserving further investigation.

### Funding

This work was supported by the National Natural Science Foundation of China (grants 41602358, 41572346) and the special project of the fundamental scientific research of the Institute of Geology, China Earthquake Administration (IGCEA1713, IGCEA1508). The funders had no role in study design, data collection and analysis, decision to publish, or preparation of the manuscript.

## Supporting information

S1 Data End-member abundances and densities of the Sikouzi grain-size sequence. (XLS)
